# From *Subliminality*, to the *Unconscious Mind*: Philosophical Lineages, Evolutionary Paradoxes, and the Future of the Origins of the Unconscious

**DOI:** 10.1007/s12124-026-09974-3

**Published:** 2026-03-17

**Authors:** Myron Tsikandilakis, Persefoni Bali, Roland Erich Uriko, Victοria-Maria Pasachidou, Romina Leonor Toranzos, Konrad Szczesniak, Christopher Raj Madan, Pierre-Alexis Mével, Alison Grant Milbank

**Affiliations:** 1 School of Psychology, University of Nottingham, Nottingham, England; 2 Medical School, Faculty of Medicine and Health Sciences, University of Nottingham, Nottingham, England; 3 School of Cultures, Languages and Area Studies, University of Nottingham, Nottingham, England; 4 Institute of Psychology, University of Tartu, Tartu, Estonia; 5 School of Science and Technology, Nottingham Trent University, Nottingham, England; 6 Estudos da Linguagem, Universidade Estadual de Londrina, Londrina, Brazil; 7 Institute of Linguistics, University of Silesia, Katowice, Poland; 8 Institute for Foreign Languages, Palacký University, Olomouc, Czechia; 9 Department of Philosophy, University of Nottingham, Nottingham, England

**Keywords:** Subliminal, Unconscious, Origins, Evolution, Differences

## Abstract

In previous works, we provided empirical evidence and the theoretical foundations for a conceptual dissociation between subliminality, such as purported emotional responses to invisible-imperceptible elicitors, and the intelligent workings of the notion of an unconscious mind, such as involuntary and automatic physiological experiences and behavioural patterns that precede but eventually interact with conscious awareness and evaluation. In this manuscript, we address this differentiation from a *Theory of Evolution* perspective. We show and discuss that a singularly subliminal module of unconscious processing could not have led to phylogenetic and ontogenetic skill acquisition, conscious problem-solving, volitional social engagement, inhibition and affect, and the development of consciously actionable cognitive-behavioural personality traits and characteristics. We argue that unconscious processing in the complete absence of conscious involvement and awareness, meta-awareness, and meta-cognition, would constitute an evolutionary hurdle and limitation. In contrast, we discuss and provide evidence that unconscious responses that occur before but eventually involve and interact with conscious awareness is a necessary condition for ecological adaptation. We highlight that unconscious-and-conscious communication, and interactions can contribute to implicit and explicit skill-acquisition, immediate physiological responses to threatening and social-related elicitors, and the ability to evaluate, appraise, re-appraise and align our experiences and responses to real-life ecological settings and adaptive demands. We conclude our manuscript with a discussion concerning the lack and need thereof of an experimental paradigm with which to, firstly, properly and appropriately explore subliminality, and, secondly, systematically and validly explore the interactions and workings of consciousness and the unconscious mind.

**Supplementary Information:**

The online version contains supplementary material available at 10.1007/s12124-026-09974-3.

## The Many *Names* of the Unconscious

In previous works, we presented the first mention of the unconscious in written language. The first mention was made sometime between 511 and 521 B.C.E by Heraclitus. In the few fragments that remain of his work, the word “ά-συνηδειτών” is mentioned, meaning etymologically “un/non-conscious” – befitting the contemporary etymology of the word – and signifying a mindless slumber, akin to sleep, that people without philosophical awareness experience in their waking hours (see Heraclitus, 2003; see also Tsikandilakis et al., 2025a; pp. 1–2). An important second mention, which altered the original meaning of the concept, was made sometime between 380 and 385 B.C.E. by Plato. In his book *Meno*, Plato describes an ageing Socrates mentioning the word “υπό-συνηδειτών”, meaning etymologically “sub/below-consciousness”, and signifying knowledge that we have and that we apply, without having explicit awareness that we do (see Plato, 2010; see also Tsikandilakis et al., 2019a; pp. 1–2). Accordingly, it is not truly a surprise that this term, that in its two very first mentions was used to signify such diverse concepts as *knowledgeless oblivion* and *oblivious knowledge*, would develop to be one of the most provocative and dividing concepts in human history (see Tallis, 2002).

As such, and consequently, the strictly empirical reader should not hope to *survive* this, otherwise, largely empirical review without *suffering* at the very least a brief further introduction to this topic involving – even if we let Sigmund Freud and psychoanalysis rest, in this manuscript (see particularly, Sand, 2013; see also Tsikandilakis et al., 2019a, 2021a, 2023a, 2025a) – prodigious and underrecognized individuals, such as Johann Fichte, Friedrich Schelling, and Eduard von Hartmann (see particularly, Hanegraaff, 2012). The contributions of these, and many other, under-recognised individuals disentangled during their time the “predicament of metonymies” that modern science has re-created for the “unconscious”, and could lead to alleviating the vices of the latter spell (see Warren., 2009; but see also Shevrin & Dickman, 1980). Being conscious of their theses can provide the semiotic insights required for properly and appropriately understanding the theory of evolution in relation to the unconscious in contemporary psychological science (Oatley, 2019).

For example, it would be wilful ignorance to persist in the overlooking of the existence and the importance of the very first systematic approaches concerning the evolution of the unconscious, and evolution and the unconscious (see Nicholls & Liebscher, 2010). Therefore, starting from the beginning, we find ourselves before the end of nineteenth century in an epistemological predicament – initiated by Georg Wilhelm Hegel (1807/2018) and further established by Immanuel Kant (1855/1999; see also Emundts, 2013). We are at that time facing the very impossibility of self-consciousness. On the one hand, Kant presented the natural world as an environment made static and inert by restricting its conception through the deterministic and mechanistic *a-priori* human faculties of cognition and sensation, and, in so doing, allowing very little space for attributing evolutionary causalities or catalyses in the natural world and the notion of the self (see Valsiner, 2013, 2019). On the other hand, Hegel by defining self-consciousness as manifested singularly through *desire*, such as the negation-assimilation of an object, and recognizing and being recognized by another self-consciousness, creates an “I” that is causally manifested by a “Not-I”. This relational-reliant dialectic does not bridge the distance from plurality to totality and, arguably, cannot offer an address beyond the-self-in-itself for another towards the-self-in-the-self as an idiomorphic phenomenology, involving a notion of the unconscious (see Beck, 1996).

As a response to these theses, Johann Fichte in his “Science of Knowledge” (1797/2012; see also Henrich, 2015) advocates the notion that in our times label subjective idealism. In his thesis, Fichte argues that as a faculty, the “I”, or “the self”, by self-positing itself as an observer of the “Not-I”, or the world, conceives that it cannot be an outer object of conceptual cognition. Therefore, the “I” self-emerges through intellectual intuition which is the emergence of self-consciousness. This process manifests as self-knowledge and occurs inevitably by being the one-unique perception available in human cognition that can perceive itself. Therefore, through intellectual self-awareness, and the proceedings of self-intuitive knowledge, self-consciousness is further than possible; it is inevitable (see Kosch, 2021). Therefore, up to this point, we have an acknowledgement of an intellectual self-consciousness, and it is exactly from this intellectual self-consciousness that Wilhelm Schelling in his “Naturphilosophie” (1800/1978; see also Ffytche, 2011), begins his most important work and is credited with igniting a philosophical “paradigm shift” (see McGrath, 2010). Schelling is credited with reintroducing the notion of the unconscious (“das Unbewusste”) in contemporary Western thinking (see McGrath, 2013). He advocates that self-intuition and self-knowledge should result in the understanding of the self as being part of a constantly moving infinite and dynamic natural world, to which the self is more than and cannot be reduced to as little as a finite faculty of observation. Therefore, a sense of inner archaic and primordial, immemorial unity emerges between finite perception and infinite experiential intuition, and leads to the inner self coming into being as an expression of the unconscious. This occurs as a synthesis in which becoming conscious of the unconscious is the passage from a reflective capacity to an expressive and productive entelechy (see Bowie, 2010).^1^ Therefore, the first contemporary philosophical system including a notion of the unconscious *comes into being*. Schelling’s “das Unbewusste” is coined as the first contemporary *name* of the unconscious (see Dufresne, 2013^)^.^2^

A philosophical system of the unconscious would be provided by Eduard von Hartmann in his “Philosophie des Unbewussten” (1869/2014; see also Gardner, 2010). In this work, Hartmann provides the first instance of a dedicated philosophical treatise on the unconscious. Hartmann *names* the unconscious “Unbewussten”, translating more accurately to “superconscious”, and conveying several qualities that this choice of terminology implies (see Gardner, 2011). Hartmann’s unconscious is a primordial and pre-ontological force that can be expressed as an idea and a material manifestation. Therefore, by process of de-evolution and induction, Hartmann believes that wherever there is entelechy, such as a materialisation in the natural world (e.g., a tree), or a conscious representation, that results in “materialisation” through psychophysical behaviour (e.g., a smile), it constitutes evidence for synthesis or catalysis with the primordial “Unbewussten”. As elegantly explained in the *Dictionary of Philosophy and Psychology* (Baldwin, 1902/1947; see also Peirce, 1903/1967), for Hartmann “…the unconscious is the absolute principle, active in all things, the force, which is operative in the inorganic, organic, and mental alike, yet not revealed in consciousness. It is the unity of unconscious presentation and will of the logical (idea) and the alogical (will). The unconscious exists independently of space, time, and individual existence, timeless before the being of the world. For us it is unconscious, for itself, it is superconscious (überbewusst)”. Here, it should be noted, that Hartmann believed that artistic expression could result from unconscious will, but that the better method for approaching the überbewusst would be a method of inductive natural science of empiricism, such as the exploration of the source (the unconscious will) through observation and reflection of its entelechic or synthetic manifestations (the natural world, or conscious-logical behaviours and ideas). With Hartmann’s system, the notion of the unconscious is, firstly, dedicatedly addressed, and secondly, linked directly to evolutionary dynamics (see Gardner, 2013).^3^

Therefore, Fichte, Platner, Schelling and Hartmann (among others), and, accordingly, the “Unbewußtseyn “, “das Unbewusste “, and the “Unbewussten” are the first *names* of the unconscious. They are the concepts that bring us from the assertion of self-consciousness to a philosophy of the unconscious, and eventually directly link the latter with evolutionary processes. These are not the only *names* of the unconscious, they are the “roads less travelled” in prelude to a discussion of the unconscious and the theory of evolution, possibly due to the analytical difficulties they involve in themselves, and to the difficulties they present to linking them within a sequential lineage of a systemic framework of ideas (see Sanderson, 2015). Further to these, the reader could only benefit from studying such pioneers as Carl Gustav Carus (Valsiner, 2024), Jacob Böhme (Gentzke, 2016), Pierre Janet (Craparo et al., 2019), Carl Lange (Lang, 1994), Karl Wilhelm Bürdach (Janssen, 2023), and related thinkers (for a review, see Gödde, 2011). These thinkers – although we obstinately and passionately seem to ignore or wilfully forget this fact – were included and discussed in William James’ (1890/1946) *Principles of Psychology*, Wilhelm Wundt’s (1873/1948) *Principles of Physiological Psychology* and Gustav Fechner’s (1860/1948) *Elements of Psychophysics* (for a review on this subject, see Schachter, 2012), which are considered the foundational texts of contemporary psychology, psychophysiology and psychophysics (see Mandler, 2011).

Furthermore, Darwin’s “On the Origin of the Species” (1859/1964; see also Smith, 1978) owes to these under-recognised individuals – to the extent that the notion of the unconscious is concerned (see particularly, Reed, 1998) – as much as contemporary psychological science owes to Darwin’s theory of evolution (see Bolhuis et al., 2011). For example, Darwin starts unfolding his notion of the unconscious as an agency expressed via phylogenetic (among species) and ontogenetic (within species, and developmentally adaptive) expression of heritable instincts; in this conceptualisation, Darwin defines an instinct as an unconscious process, and not a subliminal perception (see relevantly, von Hartmann, 1869/2014; pp. 347–371), with additional emphasis that the embodied processes, and not the environmental elicitors that trigger them, are unconscious (see Reber, 1992; Langs, 1996).

Interestingly, Darwin further connects habits to instincts, but separates their functions mechanistically. He refers to habits as recurrent patterns of behaviour and practices, which are acquired during the lifespan of an individual and performed consciously and voluntarily. Through repetition, these actions are imprinted in behavioural systems. They gradually become refined, and involve less attention and, therefore, conscious deliberation for their performance. This process leads to their transition from habits to instincts, or *hereditary habits*, that are then practised unconsciously (see Portera & Mandrioli, 2021). Darwin also emphasises the unconscious as part of an adaptable and inherited innate mechanism set in action by natural selection. The unconscious is, thus, used to additionally describe the unaware biological processes involved in the conscious apprehension of human actions (see Larson & Brauer, 2009). In this manner, he suggests an additional route of unconscious expression, namely one that through the actionable expression of heritable unconscious instincts, information emerges to consciousness as optimal behaviours, skills or technologies, that via *Descent with Modification* constitute advanced and adaptive faculties of natural selection (see Seth & Baars, 2005). Here, Darwin proposes a natural selection advantage – a sort of phenomenological entelechy (see Munz, 2002) – of unconscious action emerging to conscious knowledge and awareness, and prologues the *New Look of the unconscious mind* that advocates the evolutionary value of double-stream interactions of conscious and unconscious processes in contemporary psychological science (Baars, 1997a, b; 2003; 2007; Bargh, 2013, 2017; Bargh & Hassin, 2021). Therefore, Darwin, notwithstanding certain exceptions, such as his finality in terms of trait development, signifying here his categorical-Mendelian notions of phylogenetic and ontogenetic heritability of complex psychological traits and characteristics (Zeng, 1994), integrates the philosophical origins of the unconscious in a scientific theory of evolution (see Ludwig & Welch, 2019; see also Appendix 1.1–1.7).

We are now, therefore, in a position of having sequenced a systematic discourse of the lineage of the philosophical origins of the unconscious, and, moreover, “the missing link” from a philosophy of the unconscious to the unconscious within the framework of the theory of evolution. These, and of course the scientific and conceptual developments and frictions of the late part of the 19th, and the early part of the twentieth century, which we have discussed thoroughly elsewhere (see Tsikandilakis et al., 2019a, b, c; pp. 3–9; 2021a; pp. 2–7; 2023a; pp. 2–5; 2025a; pp. 2–14), led to the current status quo concerning the unconscious (see Northridge, 2013; Tallis, 2002). Stemming, therefore, from at least two centuries of conceptual and scientific debates and discourse, concerning whether and what the unconscious is (see Rand, 2004), we are presented in contemporary psychological science with two, frequently, but erroneously equated, and in fact conflicting, *antecedents* or *by-products* of the philosophical and evolutionary origins of unconsciousness (see Bargh & Morsella, 2008): The notion of *an unconscious mind* (see Baars, 2003; 2007; Bargh, 2013, 2017), and the notion of *the subliminal* (see Öhman et al., 2007; Warren, 2009; Brooks et al., 2012).

## The Subliminal

The word subliminal can be translated etymologically from Latin as “below the threshold”, implied here to mean “of conscious awareness” (see Elgendi et al., 2018). In contemporary psychological science, subliminal processing refers to stimuli that are attended without conscious awareness. It refers to *subliminal perception* operationalised as the *dissociation* between controlled for exhaustivity, exclusivity and dedicated null awareness of a presented elicitor (for a dedicated review of terms, see Overgaard & Timmermans, 2009; pp. 507–11), experimentally implemented via various tachistoscopic methods of visual suppression, such as backwards masking (for a comprehensive review, see Breitmeyer, 2008; pp. 9–14), that result in quantifiable emotional and behavioural changes (see Brooks et al., 2012; pp. 2963–4). The neuroscientific, and evolutionary foundations of these purported phenomena are suggested to relate to that we have retained a fast and crude archaic alarm and encoding neural system that confers adaptive survival value in response to very brief, such as 1/60th, and in some reported cases as little as 1/144th, of a second, unconsciously attended elicitors (see Meneguzzo et al., 2014; pp. 1–7). The function of this system is suggested to enable us to respond to and encode ecologically demanding information that cannot be allowed to rely on slower processes of conscious appraisal and evaluation. This system is suggested to operate via a direct subcortical neural pathway from the visual thalamus to the amygdala. In this process, this system is suggested to bypass the visual-occipital cortex, and subsequently via innervations of temporal lobe structures, such as the pulvinar and the locus coeruleus, causing the activation of the brainstem, and the downstream dissemination of peripheral physiological arousal, and/or a feedback loop to the hippocampus and the encoding of this information to *subliminal memory*, such as memory entries that are inaccessible to conscious recall, but confer motivational value for unconscious responses and actionable behavioural outcomes (see particularly, Öhman et al., 182–183; Liddell et al., 2005; pp. 234–9; Wuethrich et al., 2018; pp. 633–4 & 639–41).

Key concepts in these processes are the notions of *subliminal perception*, the *dissociation paradigm*, and the undeservedly underrated possibility of the notion of *qualitative processing differences* (see Sandberg et al., 2022). Subliminal perception has been used to suggest that there are neural pathways that can lead to the attendance of sensory stimuli in the absence of conscious awareness, meta-awareness and meta-cognition (for comprehensive review of these terms, see Tsikandilakis et al., 2020a). This process is suggested to be methodologically illustrated via the dissociation paradigm, which demands that an objective and unbiased index of perception, such as d’, A’, A’’ or A (see Zhang & Mueller, 2005) should show evidence for imperceptibility of an elicitor that nevertheless is shown to result in emotional or behavioural changes. In this process, a very understated concept is the notion of qualitative processing differences. According to our own *Theory of Physiological and Behavioural Meta-Cognition*,^4^ the notion of qualitative processing differences refers to the concept that even if an elicitor can be subliminally perceived, the emotional and/or behavioural changes which this elicitor will result to will manifest in consciousness as introspective changes in physiology, and perceptible behaviours (see Tsikandilakis et al., 2018, 2019a, 2021a).^5^

Therefore, to provide a thorough epistemological address of subliminality, in psychophysical terms, as postulated by subliminal perception and the dissociation paradigm, when perception of a stimulus is defined as α, and responsivity to a stimulus is defined as ε, then subliminal processing occurs when ε > α | α = 0. Furthermore, if we would like to include the qualitative processing differences notion and signify that subliminal exposure can result only in implicit responses, subliminal processing can be interpreted to occur when ¬ε_α_ > α | α = 0 (for a comprehensive review, see Erdelyi, 2004).^6^

## The Unconscious Mind

The notion of the unconscious mind refers to the existence of non-conscious processes that comprise behavioural guidance systems, such as perceptual and evaluative systems, that experientially, and as actionable behaviours, can precede, but eventually progress to recruit and interact, with conscious awareness and meta-cognition via a continuous two-stream communication loop of conscious and unconscious appraisals, re-appraisals and evaluations (see Baars, 2002; 2003). The original concept of the unconscious mind was predominantly focused on the precedence of action over reflection (for a review and critical discussion of this conceptualisation, see Kastrup, 2017). In recent works, we have additionally shown that automatic and involuntary central and peripheral nervous system responses to overt and perceptible elicitors contribute to ecologically necessary physiological experiences, and actionable behaviours, due to their contribution to response-accessibility, such as stimulus sensitivity, and elicitor-access, such as stimulus awareness, and can, even in the absence, and inhibition, of behavioural outcomes, experientially precede but eventually recruit and interact with conscious awareness and evaluation (Tsikandilakis et al., 2025b, c, d).

As such, the concept of the unconscious mind – termed frequently as the *new look social paradigm of the unconscious* (see Bargh, 2013) – can be considered a neo-Darwinian notion *revived* by contemporary psychological theorists and researchers to provide insights as to how the unconscious mind enfolds a system that involves intelligent, experiential and physiological correlates, in response to perceivable, but not imperceptible, emotional elicitors. This is suggested to occur to accommodate the need for both imminent reactions and the advancement of sophisticated responses to environmental demands. This could be interpreted to signify that unconscious processes interact with conscious awareness, *either/or* by emerging to consciousness in themselves, and more likely, as emergent end-products of experiential physiology, and perceptible behavior. These interactions have been suggested to comprise two correlated functions with related but not identical neuroscientific pathways and evolutionary determinations.

One function refers to unconscious-to-conscious emergence and interactions. This process refers to constitutional – phylogenetically and ontogenetically inherited – unconscious behaviors and responses that provide some evolutionary advantage. The conscious perception of these unconscious, automatic and involuntary emerging experiences and behaviors, or in the case of perceptual elicitation, conscious attendance to perceptible stimuli that cause these automatic and involuntary experiences and behaviors, can reveal their evolutionary-adaptive value. Subsequently, through double-stream interactions between conscious and unconscious processes, their perception allows us to develop experiential strategies, behavioral traits, and, furthermore, technological advancements and innovations to accommodate their improvement and continuity. The neuroscience of the unelicited-natural emergence of these processes is suggested to start with the expression of inheritable unconscious experiences and behaviors originating in temporal lobe structures, such as the amygdala, and their subsequent perceptual processing in executive-function brain structures, such as the pre-frontal cortex. In case of perceptual elicitation, a visually perceptible stimulus is presented, processed in the visual thalamus, and subsequently forwarded to the visual-occipital cortex. From there, a dorsal pathway through the parietal and a ventral pathway from the temporal lobe to the pre-frontal cortex compete for temporal primacy, with the latter largely considered to typically achieve first place in this race, therefore, showing primacy effects of unconscious experience over strictly conscious awareness, appraisal and evaluation (see Bargh, 2014; Bering & Shackelford, 2004; Goldstein & Young, 2022; Oschman & Pressman, 2013).

The notion of the unconscious mind has also been used to support the occurrence of a conscious-to-unconscious interactional skill-acquisition and performance-automatization function. In this case, extensive and dedicated repetition of a task, that demands conscious attention, such as writing and typing, driving a vehicle, engaging in a sport, or playing a musical instrument (see Sherman et al., 2014), can lead to the inception of that skill within a space of implicit performance, which is suggested to subsequently rely on automatic and unconscious mechanisms (see Jimenez et al., 2019; Ramsøy & Overgaard, 2004). In this case, therefore, conscious effort provides the evolutionary-adaptive advantage of making a repeatedly exercised task unconscious, and in so doing releases cognitive and attentional resources and, critically, co-benefits that task with the spontaneous, intuitive and highly efficient reaction, response and refinement systems of our unconscious intelligence (for a relevant review, see Tucker et al., 2022). This is suggested to take place as a reverse feedback loop from conditioned and extensive pre-frontal attention that is encoded, consolidated and, therefore, available for retrieval as an automated process, in the hippocampus, and is suggested to further interact with temporal lobe structures and the pre-frontal cortex, to constantly refine the encoding and recall of the conditioned performance (see Baars, 1997a, 1997b, 2002; 2003; 2007; Bargh, 2013, 2014, 2017; Cleeremans, 2011, 2013, 2014, 2019, 2025; Bargh & Hassin, 2021; Jimenez et al., 2025; Norman, 2010; Stamenova et al., 2018).^7^

Therefore, to provide a thorough epistemological address of the new look of the notion of an unconscious mind, in psychophysical terms, when unconscious-to-conscious interactions are involved, such as the eventual emergence to consciousness of initially unconscious, automatic and involuntary experiences and actions, this can be interpreted to signify that for τ(χ) and τ(y) being two distinct points in time, unconscious responses occur when ε_τ(x)_ ≺ α_τ(y)_ | α > 0; or if we would like to present this system in comparison to subliminality, and show the inclusivity of explicit responses stemming from initially unconscious elicitors, unconscious processes occur as (ε_τ(x)_∼α) ≺ α_τ(y)_ | α > 0. Conversely, when the concept of the unconscious mind refers to conscious-to-unconscious interactions, such as the adaptive processes of making a conscious skill automatic, this is suggested to occur as ε_(α > 0)_ **|** ε(Π) ⟹ ε_(α ≅ 0)_ (for a comprehensive review, see Bargh & Morsella, 2008).^8^

### Evolutionary Paradoxes

Of Archaic Retention, Unprecidentalism, Unbiased-Subjective Imperceptibility, and Exclusive-Exhaustive Unresponsiveness, and other *Daemons.*

Until this point in our discourse, from the philosophical origins of the unconscious, and the theory of evolution, to the notion of an unconscious mind, and with the singular exception of subliminality, we can discern a consensus: The unconscious emerges, it explicitly expresses itself, it becomes apparent, or perceptible, and it interacts with conscious functions and awareness. In philosophy, this occurs as an entelechy, such as the emergence of the unconscious in artistic expression, or the manifestation of psychophysiological behaviours and the representation of ideas, and their apperception. In the theory of evolution, it is a consciously perceptible outcome of unconscious processes that confers evaluative-adaptive value through descent via modification. In contemporary notions of the unconscious mind, it occurs – similarly to philosophy, and the theory of evolution – as an interaction among initial unconscious processes and responses, and eventual consciously perceptible cognitions, responses and behaviours, and self-awareness.

The workings of subliminal perception have been suggested to *abstain* from this consensus (see Erdelyi, 2005). This is due to that their evolutionary value is based on our ability to utilise an archaically retained subcortical pathway with survival and social adaptability value. This subcortical pathway allows us to respond to very-brief, imperceptible but unconsciously attended elicitors that require immediate adjustment to ecological demands, such as unconscious threat, and socio-emotional cues. This is the *dicitur magna* of the evolutionary advantages of subliminal processes in contemporary psychological science (see Öhman, 2013; Öhman et al., 2007). Nevertheless, several psychologists *do not see* evolutionary advantages in this description of unconscious processes, they *see* limitations (see Bargh, 2006). For example, one important discontent with subliminal processing, as defined in contemporary psychological research (see Turner, 2015), is related to the ecological validity of its experimental operationalisation (see Warren, 2009). We discussed previously that subliminal elicitors are presented for brief durations using a method of visual suppression, such as backwards masking, continuous flash suppression or binocular rivalry (for a review, see Breitmeyer, 2008). We explained that these brief presentations are shown typically for 1/60th of a second (60 Hz; 16.67 ms), and sometimes for as brief as 1/144th of a second (144 Hz; 6.94 ms). If we look more arduously at the actual meta-analyses in this subject over the last 15 years, we will see instances of emotional responses to elicitors presented for as brief as 1/244th of a second (244 Hz; 4.09 ms; see Brooks et al., 2012; pp. 2964–2965; Van den Bussche et al., 2009; pp. 462–463; Dahlen et al., 2022; pp. 2–11; Gambarota et al., 2022; pp. 7–9; Mertens & Engelhard, 2020; pp. 259–261; Meneguzzo et al., 2014; pp. 10–11; van der Ploeg et al., 2017; pp. 141–143).

The important problems with this happenstance are what we will define here as the paradoxes of *Retention and Unprecidentalism* (for a commentary on meta-terminology, see Valsiner & Brinkmann, 2016). As many psychological theorists and researchers have very strongly and critically highlighted, in this instance, we are presented with the argument that we have retained an archaic subcortical pathway to masked and brief proceedings that never occurred throughout the history of our evolution (see Baars, 2002; Bargh, 2006, 2011; Cetnarski et al., 2014; Gawronski et al., 2007; Panksepp & Panksepp, 2000; Pessoa & Adolphs, 2010; Huprich, 2011; Mudrik et al., 2024; Vadillo et al., 2016). In simple terms, during our evolution the vast majority – if not the entirety – of the elicitors we encountered were supraliminal. We were never called to respond neither to masked nor to very brief stimuli. Therefore, the current argument of subliminality stands as that we have retained an archaic neural pathway as a response to conditions that never occurred throughout our ontogenetic history and developmental lifespans. Concerningly, even if they did, they would operate subliminally and remain subliminal (see Snodgrass, 2004), therefore, they would not have allowed us to proceed through the course of our evolution to implicit an explicit skill acquisition, conscious problem-solving, voluntary social engagement, inhibition and affect, and the development of consciously actionable cognitive-behavioural personality traits and characteristics (see particularly, Bargh & Morsella, 2008; pp. 74–77).

A possible defence of at least visual suppression in this argument is that it is used to simulate subliminal/unconscious perception (see Bob, 2003; Dehaene, 2004; Dehaene et al., 2006; Frumento et al., 2024; Hung & Hsieh, 2015; Öhman, 2013; Öhman et al., 2007; Overgaard & Timmermans, 2008). Nevertheless, this argument too fails the methodological trial by ordeal (see particularly, Yu, 2023; pp. 432–443). As mentioned previously, the requirements for subliminal perception are that it results in responses that are exhaustive, exclusive, and dedicated. Exhaustive-response analyses, or exhaustiveness, means that all types of responses, such true positives (TP), true negatives (TN), false positives (FP) and false negatives (FN), should be explored for emotional outcomes (for a review of terminology, see Macmillan, 2002). Accordingly, exclusive responses, or exclusivity, means that responses to subliminal perception should not occur for elicitors that were not perceived subliminally, such as overt elicitors (TPs), or responses to TNs and FPs. Finally, having dedicated responses means that subliminal perception should result in responses only for elicitors that were subliminally perceived, such as FNs, signifying here that an emotional elicitor that was presented was not detected or recognised by a participant (see particularly, Overgaard & Timmermans, 2009; pp. 504–511).

In our research, we have provided refutations for these tenets and requirements for subliminality, and subliminal perception. We applied a model for subjective subliminality/unconsciousness, we applied Bayesian analyses, based on minimum effects sizes of interest characteristics (see Dienes, 2016, 2019, 2021, 2025; see also Appendix 3) to define, using sensitivity index A (Zhang & Mueller, 2005; for a mathematical review concerning the choice of this metric, see Tsikandilakis et al., 2025a; pp. 69–73), what duration for each stimulus type and each individual results in null perception under condition of visual masking (for relevant reviews and tutorials on this method, see Tsikandilakis et al., 2019a, 2020a, 2021a, 2023a, 2025a). Subsequently, we used these subjective/individual per participant and stimulus-type thresholds to measure CNS, and PNS, and self-reports to emotional faces showing basic emotions (Tsikandilakis & Chapman, 2018; Tsikandilakis et al., 2018, 2020c, 2025b, c), emotional scenery, such as IAPS stimuli (see Lang et al., 1997; see also Branco et al., 2023), including also depictions of immoral behaviour (Tsikandilakis et al., 2021c), scramble and semantic lexical tasks (Tsikandilakis et al., 2020b), attractive faces and images (Tsikandilakis et al., 2019b, 2025d), cultural-dialects of emotion (2019c; 2021b; 2023b), and even non-basic, and previously unexplored emotional states, such as hostility (2020b), and melancholy, misery, bereavement, and despair (2023c). For unbiased null perception defined as α_(Un.)_ = 0, when using Bayesian analysis, and signal detection theory, and for ε being any kind of response to these truly subliminal stimuli, we reported in every single instance of our work Bayesian evidence for the null (B <.3), for every type of response and stimulus type (Tsikandilakis et al., 2025c). When we implemented our method for subjective/individual unconsciousness using hit-rates, that can be biased by participant response strategies (see Stanislaw & Todorov, 1999; see also Macmillan, 2002), and then various static durations of presentation as per previous research (6.94 to 50 ms; see) we, respectively, started reporting minor effects and replicated the effects reported in previous research for TPs and FPs only (see Brooks et al., 2012; Van den Bussche et al., 2009; Dahlen et al., 2022; Gambarota et al., 2022; Mertens & Engelhard, 2020; Meneguzzo et al., 2014; van der Ploeg et al., 2017).

This could be interpreted to be *a manifesto of refutation of subliminal processes*. It is not. It is far more complex than that, and we had to start with philosophy, and continue to the theory of evolution, and then to contemporary science to understand why. With our model, we confirmed what scholars and researchers from philosophy, evolutionary theory, and psychological science have theorised (see particularly, Snodgrass, 2004). We empirically illustrated an idea over centuries old (Fichte, 1797/2012), that has been discussed dedicatedly in psychological science, though, unfortunately, mostly in vain (see Erdelyi, 2003), for the past 128 years (see Sidis, 1898): We cannot experience or behave due to unconscious process, and/or subliminal elicitors, without consciously perceiving ourselves as having an experience and acting a behaving (see Hernández-Gutiérrez et al., 2025; Erdelyi & Zizak, 2003; Klein et al., 1958; Moore, 1988; Pessoa et al., 2005; Pessoa et al., 2006; Szcześniak, 2024; Szcześniak & Řeřicha, 2024; Yu, 2023; Warren, 2009; Wiens, 2006; Williams Jr, 1938).

Let us properly and appropriately understand this “olde” paradox as best as we can, so we can bridge the past and the future of the unconscious and the subliminal. As we discussed above, in our research, when we used hit-rate thresholds for subjective individual unconsciousness, and static durations of presentation, we showed various response effects for TPs, such as correctly reporting that a stimulus was presented, and for FPs, such as incorrectly responding that as stimulus was presented. We are very grateful to have received excellent collegial responses as to the former (TPs) outcome, for showing that the exhaustive, exclusive and dedicated exploration of truly subliminal elicitors does not result in participant responses. We do believe that this is an important finding, but that the real unriddling of the mysteries of the subliminal could lie with our reporting response effects for FPs (Tsikandilakis et al., 2018, 2019a, 2020a). This could be interpreted to signify that when participants either experienced something or perceived a change in behaviour, they responded seeing a visually suppressed elicitor in the absence of an elicitor. In simple words, perception of an elicitor (TPs), or self-perception of maybe subliminally induced arousal, or maybe noise arousal due to a host of effects we have described elsewhere (Tsikandilakis et al., 2020a), resulted in responses for having seen a stimulus, even when a stimulus was not presented.

This finding has been shown before in seminal publications, but it seems that it has been somewhat lost to time in the larger context of the more nuanced with the TPs vs FNs controversies of the *subliminal wars* (see for example, Pessoa et al., 2005, 2006). Therefore, as proof of concept that this phenomenon does occur, and, additionally, that it is not an artifact of our own multifaceted method of subjective/individual consciousness and unconsciousness (Tsikandilakis et al., 2023a), we recently attempted to show this phenomenon as it would occur in a static duration study (see Tsikandilakis et al., 2025c; pp. 1–5 & 7–8). We showed that based on previous studies using faces, the most common duration for backward masking was 16.67 ms (see Brooks et al., 2012; Gambarota et al., 2022; Meneguzzo et al., 2014; Mertens & Engelhard, 2020; van der Ploeg et al., 2017). For this static duration, for an exhaustive analysis (TPs, TNs, FPs & FNs) using the coder functions of G-power (Faul et al., 2009; see also https://clincalc.com/stats/samplesize.aspx) and calculating per response and stimulus-type trial-contour sequence estimators (see Baker et al., 2021; see https://shiny.york.ac.uk/powercontours/), we showed that an n = 193 and trial-repetitions per stimulus type (fearful, angry, happy, sad, neutral & pattern blurs), set at k (6) = 200 would be required to achieve a statistical power of P_(1− β)_ ≥.9 (η^2^_p_ =.1; p ≥.01; f ≥.1; d ≥.2; *p* ≤.05; P _(H01/10)_ ≥.9; B <.3; B > 3) (see Giner-Sorolla, 2019, pp. 6–11; Kelter, 2021, pp. 5–14; Dienes, 2021, pp. 4–17).

The results of our study are adapted, with permissions from the original publication, in Table 1. using carefully punctuated NHST and Bayesian analyses, and exact effect sizes, for every single stimulus and response type. The responses make an important argument: Where there is arousal, or the perception or apperception of arousal, or, a behaviour, or the perception or apperception of a behaviour, participants respond as Fichte, Plankter, Schelling, von Hartman, and Darwin, and Baars, Bargh, Erdelyi, Morsella, Pessoa, and so many others, theorise: They assume conscious awareness of a triggering condition. In even simpler terms: Even if unconscious/subliminal perception occurs when it involves consciously perceptible responses these responses cannot remain subliminal; they are perceptible and reported. Subliminal perception as a report of lack of awareness of an elicitor that produces physiological or behavioural changes is inconsistent.

**Table 1:** Illustration of exclusive, exhaustive and dedicated analyses

Mean (SD)					
Α		Pre-Stimulus		Post-Stimulus	
		SCR	HR	SCR	HR
Fear	TP	.006 (.003)	.993 (.114)	.075 (.013)*	5.44 (.778)*
	**FP**	.029 (.006)*	3.038 (.596)*	.072 (.017)*	5.346 (.787)*
	TN	.005 (.003)	1.017 (.127)	.011 (.003)	1.116 (.112)
	*FN*	*.005 (.002)*	*1.011 (.117)*	*.01 (.004)*	*1.107 (.12)*
Anger	TP	.006 (.003)	1.001 (.115)	.065 (.012)*	3.32 (.646)*
	**FP**	.019 (.006)*	2.747 (.407)*	.054 (.008)*	2.939 (.537)*
	TN	.006 (.003)	1.021 (.128)	.012 (.004)	1.11 (.118)
	*FN*	*.004 (.003)*	*1.003 (.116)*	*.011 (.004)*	*1.08 (.11)*
Happy	TP	.006 (.003)	.998 (.113)	.066 (.013)*	3.41 (.623)*
	**FP**	.019 (.005)*	2.778 (.402)*	.053 (.008)*	2.902 (.523)*
	TN	.005 (.003)	1.024 (.125)	.011 (.004)	1.107 (.119)
	*FN*	*.004 (.003)*	*1.009 (.123)*	*.011 (.004)*	*1.126 (.121)*
Sad	TP	.004 (.002)	.989 (.115)	.021 (.003)	1.099 (.113)
	**FP**	**.006 (.003)**	**1.007 (.115)**	**.013 (.003)**	**1.112 (.12)**
	TN	.006 (.003)	1.014 (.118)	.011 (.003)	1.108 (.119)
	*FN*	*.004 (.003)*	*1.006 (.118)*	*.012 (.004)*	*1.109 (.116)*
Neutral	TP	.006 (.003)	1.001 (.114)	.012 (.004)	1.1 (.108)
	**FP**	**.005 (.003)**	**1.004 (.118)**	**.011 (.003)**	**1.101 (.12)**
	TN	.004 (.003)	1.047 (.13)	.012 (.004)	1.109 (.119)
	*FN*	*.004 (.002)*	*1.003 (.116)*	*.013 (.004)*	*1.111 (.122)*
Standardised Cohen's d (BFs)					
Β		Pre-Stimulus		Post-Stimulus	
		SCR	HR	SCR	HR
Fear	TP	.079 (.23)	.155 (1.37)	4.752 (+ ∞)*	5.993 (+ ∞)*
	**FP**	4.456 (+ ∞)*	4.354 (+ ∞)*	3.543 (+ ∞)*	5.772 (+ ∞)*
	TN	.133 (.97)	.123 (.86)	.097 (.29)	.111 (.76)
	*FN*	*.154 (1.37)*	*.134 (.97)*	*.092 (.29)*	*.109 (.76)*
Anger	TP	.074 (.23)	.146 (1.31)	4.018 (+ ∞)*	3.207 (+ ∞)*
	**FP**	2.383 (+ ∞)*	5.183 (+ ∞)*	3.83 (+ ∞)*	2.335 (+ ∞)*
	TN	.079 (.23)	.119 (.79)	.093 (.29)	.11 (.76)
	*FN*	*.187 (2.71)*	*.143 (1.25)*	*.09 (.29)*	*.114 (.79)*
Happy	TP	.081 (.23)	.149 (1.26)	3.865 (+ ∞)*	3.047 (+ ∞)*
	**FP**	2.727 (+ ∞)*	5.338 (+ ∞)*	3.69 (+ ∞)*	2.296 (+ ∞)*
	TN	.133 (.97)	.116 (.81)	.093 (.29)	.112 (.79)
	*FN*	*.187 (2.71)*	*.134 (.97)*	*.092 (.29)*	*.106 (.76)*
Sad	TP	.113 (.76)	.159 (1.37)	.18 (.2.71)	.111 (.78)
	**FP**	**.074 (.23)**	**.137 (.97)**	**.097 (.29)**	**.108 (.76)**
	TN	.075 (.23)	.126 (.89)	.094 (.29)	.109 (.76)
	*FN*	*.187 (2.71)*	*.139 (.97)*	*.091 (.29)*	*.11 (.76)*
Neutral	TP	.075 (.23)	.146 (1.31)	.093 (.29)	.112 (.79)
	**FP**	**.013 (.09)**	**.141 (1.31)**	**.094 (.29)**	**.109 (.76)**
	TN	.078 (.23)	.089 (.27)	.094 (.29)	.113 (.79)
	*FN*	*.116 (.81)*	*.143 (1.41)*	*.095 (.29)*	*.108 (.76)*

Tab 1: In **A**. Mean and SD for SCR and HR. In **B.** standardised effect size Cohen’s d and Bayes Factors. Asterisks (*) show significance at *p* ≤ .001, and plus infinity (+ ∞) values for Bayesian analyses. Underlined text shows TPs, such as correctly recognising an elicitor. **Bold** text shows FPs, such as incorrectly responding recognising an elicitor. *Italic* text shows FNs, such as incorrectly responding not recognising an elicitor. TPs and FNs showed evidence for physiological responses, while FNs did not show any evidence for responses. These findings challenge the feasibility of reporting physiological and behavioural responses to subliminal perception (adapted with journal permissions under open-access dissemination policy from Tsikandilakis et al., 2025c; p. 14)

## The Future of the Origins of the Unconscious

We have come to this point in our discussion, from philosophy and the beginning of the theory of evolution to contemporary psychophysics and biostatistics, hoping to have highlighted the importance of history for understanding contemporary science, and to be able to contribute *our vote* on how to conceptually interpret and experimentally explore subliminal perception and the notion of the unconscious mind. Our opinion is that the current paradigm for subliminality advocates the ecological validity of conditions, such as visual suppression and very brief presentations, that do not have ecologically valid evolutionary precedence. It also prohibits conscious-and-unconscious interactions which we believe have provided great phylogenetic, ontogenetic and developmental benefits throughout the course of our evolution. Finally, we believe that we have described and supported with a variety of references the applied-empirical discontent between unconscious processes and subliminal responses. In this occasion, therefore, we will re-emphasise the concept of qualitative processing differences. We argue that if we would like to test the possibility of subliminality we require subliminal perception resulting to subliminal responses. This a direly overlooked requirement discussed in relevant research (see Kiesel et al., 2008; Pessoa & Adolphs, 2010; Snodgrass, 2004; Warren, 2009).

Perhaps one way through this methodological labyrinth is ambivalence (see Valsiner, 2006). Several researchers in the last twenty years have shown implicit preferential long-term-memory engagement for images that were previously co-presented with a stimulus that was in miss-match with one of their perceptible characteristics (e.g. shape, colour, posture, gender, age), or paired with a stimulus that differed from the pairing norms of a particular trial sequence. This has purportedly happened in absence of perceptible physiological arousal, and particularly in cases of priming while distracting participants conscious-attentional resources from the perception of their own behaviour via the implementation of main-task-irrelevant parallel tasks (see Berger et al., 2019; Petty et al., 2012; Schneider & Schwarz, 2017). If imperceptible stimuli can be applied in these experimental paradigms and replicate these findings, and particularly, as we have argued before show evidence for ambivalent behaviour in non-perceptible processes, and not outcomes of processes, we could be nearing a pathway from subliminal perception to subliminal responses and their testing, at least as a non-singular evolutionary important unconscious mechanism (see Tsikandilakis et al., 2019a; see also Tsikandilakis et al., 2020c). The important task here is that we should strive for subliminal perception to result in measurable subliminal outcomes, preferably without completely compromising its evolutionary value (see Öhman et al., 2007), and we might just be able to provide the current methodological requirements for this line of research (see Erdelyi, 2005).

**Fig. 1:**
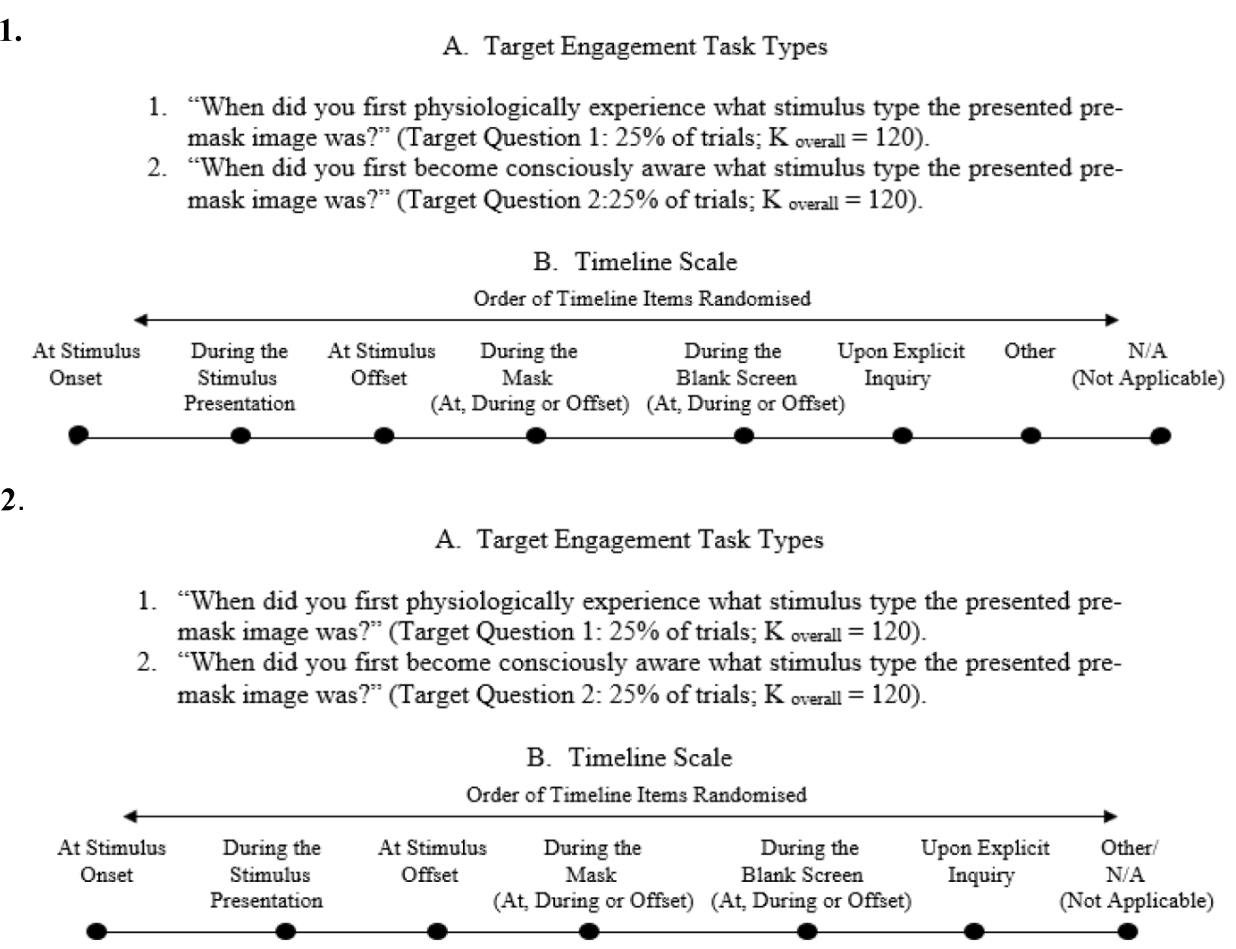
Temporal primacy assessment. Rasch analyses adapted temporal-primacy assessment self-reports. In **1**. the responses are adapted for temporal-primacy assessment of overt emotional faces presented for one second (Tsikandilakis et al., 2025b; pp. 21–3). In **2.** the responses are adapted for the temporal-primacy assessment of attractive faces presented for one second (Tsikandilakis et al., 2025d; pp. 41–3)

On the other hand, if we submit to that the current experimental paradigm for exploring subliminality is contentious, we should also submit that the current experimental paradigm for exploring the workings of the unconscious mind is incomplete (for a comprehensive review, see French & Cleeremans, 2002). In the case of the unconscious mind, we do not need alterations; we need a consensus for a valid paradigm. There are arguably some experimental methods showing the pre-Darwinian notion of successfully making a skill or an engagement implicit or unconscious mainly by exercising motor skills while performing irrelevant conscious-demanding tasks (see Cleeremans, 2013; Cleeremans & Jiménez, 2013; Greenwald et al., 2017; Tucker et al., 2022). This is important but it does not address the main claim of the notion of the unconscious mind: That unconscious responses precede, but eventually involve conscious awareness. We have made some little progress towards a paradigm for exploring this possibility. We have used Rasch analyses (see Boone et al., 2014; Boone, 2016; Boone, 2020) to distil the most reliable self-report assessments of temporal-primacy between experience and consciousness, and we present a preliminary application of our endeavours in Fig. 1 (see Tsikandilakis et al., 2025b, 2025d). We accept the debates relating to the limitations of self-reports (see Bond et al., 2021; Haralabopoulos et al., 2020; Leong et al., 2024; Leveridge et al., 2024; Mével et al., 2025; Yu et al., 2022), and we do propose that our illustrated methodology could benefit from high-temporal resolution neuroscientific techniques such as functional magnetic resonance imaging (fMRI), electroencephalography (EEG), and functional near infrared spectroscopy (fNIRS; see Dehaene & King, 2016; Denton et al., 2009; He & Raichle, 2009; Wolff et al., 2019).

## Conclusion(s)

From the philosophical beginnings of its conceptualisation, the unconscious was considered a constitutional and compelling agency or will or drive and, eventually, in evolution theory, an instinct that can exert influence in our experiences and behaviours. Since its philosophical beginnings, and in the theory of evolution, the unconscious was defined as having its *entelechy* in emerging into consciousness, and conferred advanced adaptation value for emerging into consciousness, respectively. These early conceptualisations led to two different notions of the unconscious in contemporary psychological science: Subliminality, such as responses to imperceptible or invisible elicitors, and the unconscious mind, such as automatic and involuntary experiences and actions, that precede, but eventually interact and recruit conscious awareness. We argued that subliminality as a module that involves singularly unconscious processes presents significant reportability hurdles and limitations. Conversely, we contended that subliminality as a function that does not interact with conscious awareness is paradoxical from an evolutionary perspective because it could not have led to conscious skill-acquisition and problem-solving, voluntary social engagement, inhibition and affect, and the development of consciously actionable cognitive-behavioural personality traits and characteristics. We proposed that the future of unconscious research could be better served by experimental paradigms that conceptualise subliminal perception as a non-singular unconscious module that could result specifically to subliminal responses, and additionally engage with the many evolutionary advantages of the notion of an unconscious mind that relate to conscious and unconscious syntheses and interactions.

## Supplementary Information

Below is the link to the electronic supplementary material.

Supplementary file1 (DOCX 40 KB)

## Data Availability

All data and materials have been made available at [https://osf.io/75c8u/overview](https:/osf.io/75c8u/overview) and [https://osf.io/hfyjw/overview](https:/osf.io/hfyjw/overview).

## Footnotes

1 It should be noted that Schelling’s philosophy involved no less than three distinct ideological epochs, and was a system of ideas that were constantly “underway”; always becoming, being revised and being revisited (Fisher, 2017). This created tensions between Schelling and other thinkers, such as Hegel and Nietzsche, who considered Schelling’s philosophy recurrently incomplete and constantly inconsistent (Dews, 2022). These frictions are well-documented in published lecturing disputes, and published and unpublished papers and correspondences among Schelling and several other thinkers (for a thorough presentation, complete open-source references to this bibliography, and a detailed review, see Linker, 2000).

2 Some historically nuanced academics (Hansen, 2018; Hendrix, 2015; Pecere, 2020), have proposed that the first mention of the unconscious was made by Ernst Platner in his “Philosophical Aphorisms” (1776/1949; see also Otabe, 2019). In his thesis, Platner proposes – based on Leibnitz’s *Monadology* (1714/1969; see also Garber, 2009) – that we can experience *petites*/minute perceptions that unknowingly affect our behaviour and cognition. This is based on Leibnitz’s theory that if infinite deduction leads to god, then infinite induction should lead to invisible and indivisible particles that comprise god. Leibnitz calls these particles “Monads “ (“Monade”), and considers the Monads, and particles of regressively inductive proximity to the Monads, to be imperceptible but effective elicitors, that can affect our behaviour and cognition. Platner is generally considered to simply coin the name “Unbewußtseyn “ to this process, the latter translating etymologically to “unbelievable knowing/un-conscious-being “, and more proverbially as unconscious, or unconsciousness. Beyond considering the thesis that this could, indeed, be the first *name* of the unconscious, we propose that Platner’s work is gravely under-appreciated in this occasion. In his thesis, Platner elaborates that since the self cannot be fully known to itself as a subject of self-perception, every expression and perception emerges as a representation. A representation cannot stem from knowledge of the self, and self-consciousness, it can only emerge from an “unprethinkable “ vast and unexplored innerness and therefore from the unconscious, and that this occurs irrespectively of whether we are discoursing a representation of the perception of an object, or the expression of a fantasy, or the “unconscious emergence of a drive or instinct “. These concepts are presented 119 years before Breuer and Freud claim them for psychoanalysis in “The Studies on Hysteria “ (Breuer & Freud, 1895/2001), and, moreover, in a passage that is so provocatively different to the *zeitgeist* of its time that Platner proceeds to finish his argument by quoting verbatim that “… if the reader is offended by this argument, they should read footnote 113 “. Surprisingly, nevertheless, in footnote 113, the nuanced reader discovers that Platner simply quotes vaguely and mysteriously perhaps depriving his thesis of systematic philosophical value, and semiosis, and possibly inviting obscurity and unclearness, meddling his philosophy with metaphysics (see Nicholls & Liebscher, 2013), the phrase “Die Nerven vereinigen sich allenthalben mittelfst ihrer Äefte” (“The nerves unite everywhere by means of their branches”; Platner, 1776/1949; pp. 9–12 & 39).

3 Although it would be quite unfair to consider any of the discussed concepts as proto-psychoanalytic, on the grounds that they philosophically describe, instead of therapeutically applying a model of the workings of the unconscious (see Hammer, 2007), if we did attempt an approximation of concepts, “das Unbewustte “ and the “Unbewußtseyn” point through unconsciousness, and its entelechy in conscious conception and expression, to Freudian concept of repression, and the Tally argument, such as the expansion of the ego over the id. On the other hand, the “überbewusst”, being conceived as *unconscious* within, and pre-existing as *superconscious* before and beyond the self, points closer to the Jungian notion of the collective unconscious; i.e., a universal pre-ontogenetic, yet ontogenetically inherited, and preternatural unconscious force (see, Darnoi, 2012). Additionally, if we would like to submit further comparisons, it should be noted that all three accounts of the unconscious as presented above differ to the Kantian *a-priori* in that they are philosophical analyses of the description and phenomenology of the unconscious, as opposed to the inherent limitations of our conscious or unconscious representations of the world, They also differ from Hegelian thought in that in these concepts self-consciousness has unilateral self-agency, and is not defined by proxy, via desire, or acknowledgement. Finally, it should be noted that among them, these concepts differ in that Hartmann’s “überbewusst” is defined to have its entelechy, not strictly in self-consciousness, and the expression of ideas and art, but critically, in the “scientific” observation and exploration of the natural world as involving in the manifestations of the material the transcendental entelechy of the immaterial, primordial and pre-ontological will of the “überbewusst” (see Gardner, 2013).

4 Our theory should be treated as little more than the experimental application and empirical validation of the notions presented in the origins of the unconscious, and the theory of evolution, as discussed in a previous section of this manuscript (for a comprehensive overview, see Seth & Baars, 2005).

5 As such, it is worth considering that, although, contemporary psychological science has taken less interest than it should in this phenomenon, subliminal perception “through the masking glass” of qualitative processing differences, could be better served if assessed via implicit outcome measures, such as influenced preference and non-preference of neutral items that were primed with positive and negative subliminal stimuli respectively (see Schacter, 1992), the purpose of which would be to avoid the meta-cognitive processing of emotional and behavioural end-process explicit action and affect (see Dehaene et al., 2006; Warren, 2009; Wiens, 2006; but see also, particularly, Snodgrass, 2004; Pessoa & Adolphs, 2010).

6 “|”: Mathematical symbol signifying “for”; “¬”: Mathematical symbol signifying “not related to”; “¬x_y_”: Mathematical sequence signifying “X not related to y”.

7 Although for many psychologists the notion of an unconscious mind confers higher ecological validity compared to the notion of a module of singularly subliminally unconscious processing (see Bargh & Morsella, 2008), the significant problem with the concepts it involves arises in their exploration, and the lack of a consensual and validated experimental paradigm, or set of experimental paradigms, for their exploration (see the next sections of the current manuscript; for comprehensive reviews, see Cleeremans, 2005; Jimenez et al., 2025; Norman, 2010; Stamenova et al., 2018; see also Appendix 1.1–2.1–2.2).

8 “≺”: Mathematical symbol signifying “preceding to”; “∼”: Mathematical symbol signifying “related to”; “Π”: Mathematical symbol signifying “exponential or fixed trial repeat”; “⟹”: Mathematical symbol signifying “results to”; “ ≅ ”: Mathematical symbol signifying “equal, or approximately equal, to”.
