# Supplementary material for: From Subliminality, to the Unconscious Mind: Philosophical Lineages, Evolutionary Paradoxes, and the Future of the Origins of the Unconscious
Source: Integr Psychol Behav Sci. 2026 Mar 17;60(2):27. doi: 10.1007/s12124-026-09974-3 (PMC12996011; doi:10.1007/s12124-026-09974-3)
Supplement: Supplementary file 1 — Supplementary file1 (DOCX 40 KB) [file 12124_2026_9974_MOESM1_ESM.docx]

Appendix

1. A Comprehensive Review of Darwin’s Theory of Evolution

1.1: Darwin's Unconscious: Anticipating Unconscious-to-Conscious Integration

Darwin's deployment of the "unconscious" throughout "On the Origin of Species” (1859) and “The Variation of Animals and Plants Under Domestication” (1868) anticipates the distinction central to the aims of the present paper. Specifically, Darwin's "unconscious" never denotes processing that remains forever inaccessible to awareness, such as the purely subliminal. Rather, Darwin consistently describes processes that operate without deliberate intention, but whose effects eventually manifest in consciously observable, evaluable, and modifiable outcomes (see Gregory, 2009; Sloan, 2019). This pattern recurs across five distinct usages in Darwin's work: Unconscious selection in domestic breeding, unconscious modification through correlation of growth, naturalists unconsciously seeking genealogical classification, unconscious competition in the struggle for existence, and unconscious processes in the modification of instinct. Each usage supports the theoretical position that evolutionarily adaptive unconscious processing is characterized by unconscious-to-conscious integration rather than permanent subliminality (cf. Reber, 1992; Bargh & Morsella, 2008).

1.2: Unconscious Selection: Modification Without Intention, Outcomes with Awareness

Darwin's most systematic treatment of the unconscious appears in Chapter I of the *Origin of the Species* ("Variation Under Domestication"). There he establishes a fundamental distinction between two modes of artificial selection (see Richards, 1987; Secord, 1981). Methodical selection denotes deliberate, goal-directed breeding “whereby eminent breeders try by methodical selection, with a distinct object in view, to make a new strain or sub-breed, superior to anything existing in the country” (Darwin, 1859; p. 34). Professional breeders like Bakewell and Collins exemplified this approach, greatly modifying, even during their own lifetimes, the forms and qualities of their cattle through systematic, intentional effort directed toward predetermined standards.

Unconscious selection, by contrast, “…results from trying to possess and breed from the best individual animals without any wish or expectation of permanently altering the breed” (Darwin, 1859; p. 34). Therefore, there Darwin provides his canonical illustration: "Thus, a man who intends keeping pointers naturally tries to get as good dogs as he can, and afterwards breeds from his own best dogs, but he has no wish or expectation of permanently altering the breed. Nevertheless, I cannot doubt that this process, continued for centuries, would improve and modify any breed" (Darwin, 1859; p. 35). In *Variation* *Under Domestication*, Darwin formalized this definition: "Unconscious selection is that which follows from men naturally preserving the most valued and destroying the less valued individuals, without any thought of altering the breed" (Darwin, 1868, Vol. II, p. 193).

Darwin explicitly states that unconscious selection is "more important" than methodical selection for understanding evolutionary processes (Darwin, 1859, p. 34). This prioritization is theoretically significant for current paper (see Heiser, 1988; Zohary, 2004). If dramatic modifications can accumulate through processes entirely lacking conscious planning - where breeders achieve transformative results they neither intended nor foresaw - then adaptive change requires no deliberate design. Yet crucially, the changes produced by unconscious selection do not remain subliminal. They surface into the observable world: King Charles's spaniel "…has been unconsciously modified to a large extent since the time of that monarch…" (Darwin, 1859; p. 35); the English pointer "…has been greatly changed within the last century…" (Darwin, 1859; p. 35); two flocks of Leicester sheep, both maintained with conscious fidelity to original stock, nonetheless diverged until "…they have the appearance of being quite different varieties…" (Darwin, 1859; p. 36). The breeders were unaware of the cumulative modification they effected, yet the outcomes became consciously observable, subject to evaluation, comparison, and further selection. Unconscious selection thus exemplifies the unconscious-to-conscious integration pattern: Processing without awareness of cumulative effects, but effects that eventually manifest as conscious phenomena (cf. Dehaene et al., 2006).

Darwin extended this concept beyond professional breeders to encompass all human cultivation throughout history. Unconscious selection was practiced by "…savages, ignorant of the rules of inheritance…" who, facing "…famines and other accidents nonetheless ensured that preferred individuals would leave more offspring than the inferior ones…" (Darwin, 1859, pp. 36-37). Darwin observed that "the varieties kept by savages have more of the character of true species, than the varieties kept in civilized countries" (Darwin, 1859; p. 37) - precisely because selection operated through survival pressures rather than aesthetic preferences, producing modifications of greater adaptive significance (see Diamond, 2002; Harlan, 1992). Even without any theoretical understanding of heredity or selection, these populations affected evolutionary changes that surfaced into the observable, experiential world. The unconscious process generated conscious-level outcomes.

1.3: Unconscious Modification Through Correlation of Growth

Darwin introduces a second usage of "unconsciously" that addresses unintended by-products of selection. When humans select for one trait, they "…almost certainly unconsciously modify other parts of the structure, owing to the mysterious laws of the correlation of growth…" (Darwin, 1859; p. 12). Darwin provides extensive empirical examples: "…Cats with blue eyes are invariably deaf…"; "…hairless dogs have imperfect teeth…"; "…pigeons with feathered feet have skin between their outer toes…"; "…pigeons with short beaks have small feet, and those with long beaks large feet…" (Darwin, 1859; p. 12).

A breeder selecting long beaks in pigeons does not intend to modify foot size, has no awareness that foot size is changing, and would likely be surprised to learn of the correlation (see Pigliucci, 2001). Yet the modification occurs nonetheless - a mechanistic consequence of developmental links that the breeder neither understands nor controls. The correlated changes are unconscious in their production but not subliminal in their existence: They manifest in observable phenotypes, available for subsequent conscious evaluation. What appears to be integrated design (proportional beak and foot size) is an unintended by-product of selection directed elsewhere. Complex coordinated modifications arise without conscious planning for their production - yet they enter the observable world and further selection, conscious or unconscious, can act upon them. The unconscious process feeds into conscious-level reality (cf. West-Eberhard, 2003).

1.4: Naturalists Unconsciously Seeking Genealogical Classification

In Chapter XIII of the Origin of the Species, Darwin advances a striking epistemological claim that has attracted considerable scholarly attention (see Hodge, 1977; Lennox, 2005). Pre-Darwinian naturalists, operating within creationist frameworks and consciously seeking to uncover a divine design in natural order were tracing evolutionary relationships without knowing it:

*All true classification is genealogical; that community of descent is the hidden bond which naturalists have been unconsciously seeking, and not some unknown plan of creation, or the enunciation of general propositions, and the mere putting together and separating objects more or less alike.* (Darwin, 1859; p. 420*)*

Darwin elaborates: "Descent has universally been used in classing together the individuals of the same species, though the males and females and larvae are sometimes extremely different... may not this same element of descent have been unconsciously used in grouping species under genera, and genera under higher groups?" (Darwin, 1859; p. 425). He concludes: "I believe that this element of descent is the hidden bond of connection which naturalists have sought under the term of the Natural System" (Darwin, 1859; p. 433).

This is particularly relevant to the present theoretical framework (cf. Baars, 1997; Shevrin & Dickman, 1980). The naturalists' unconscious methodology was not subliminal in the sense of remaining forever below awareness and inaccessible to conscious cognition. It surfaced in their taxonomic judgments - the classifications they produced, debated, refined, and published. Their conscious theoretical commitment was to special creation; their unconscious practical methodology traced common ancestry. The unconscious processes guided conscious scientific activity, producing outcomes that were publicly observable and subsequently available for Darwinian reinterpretation. Darwin's argument depends on the unconscious processing not remaining sealed from consciousness: It manifested in the naturalists' work, where it could be recognized and theoretically integrated. This represents unconscious-to-conscious integration operating at the level of scientific cognition itself (see Reber, 1992).

1.5: Unconscious Competition in the Struggle for Existence

Darwin employs the term "unconsciously" to describe how organisms compete in the struggle for existence. In Chapter III of the Origin of the Species, he notes that individuals "…must compete, albeit unconsciously, for what little food there is…" (Darwin, 1859; p. 62). This emphasizes that natural selection requires no awareness, intention, or purpose on the part of organisms (see Dennett, 1995; Mayr, 1982). Plants do not decide to grow toward light; animals do not intend to outcompete rivals. The struggle for existence proceeds without the combatants being aware that they are engaged in it.

Yet, the outcomes of unconscious competition are again not subliminal. Differential survival and reproduction constitute the environmental pressures to which conscious and unconscious processes must adapt. The unconscious competition shapes the observable world within which organisms navigate, perceive, and respond. Darwin writes: "A grain in the balance will determine which individual shall live and which shall die" (Darwin, 1859; p. 489) – he describes a mechanical process, yet one whose consequences pervade the experiential reality of surviving organisms. The competition is unconscious; its effects are not. Organisms that survive unconscious competition then consciously (or through further unconscious processing) navigate the world that competition has shaped. The unconscious processes feed into conscious-level ecology (cf. Cosmides & Tooby, 1994).

1.6: Instinct: Cognition That Does Not Know Itself

Darwin's treatment of instinct most directly anticipates the unconscious-to-conscious integration model (see Richards, 1987; Oatley, 2019). In Chapter VII of the Origin of the Species, Darwin compares instinct with habit, observing: "How unconsciously many habitual actions are performed, indeed not rarely in direct opposition to our conscious will!" (Darwin, 1859; p. 234). He argues that instincts have been modified "…partly by habit, and partly by man selecting and accumulating during successive generations, peculiar mental habits and actions... In some cases, compulsory habit alone has sufficed to produce such inherited mental changes; in other cases. compulsory habit has done nothing, and all has been the result of selection, pursued both methodically and unconsciously" (Darwin, 1859; p. 243).

Darwin characterizes instinct as rational action that has become habitual and heritable: *A cognitive process that does not know itself as such* (see Oatley, 2019). The hive-bees construct geometrically optimal hexagonal cells, yet "…the bees, of course, no more knowing that they swept their spheres at one particular distance from each other, than they know what are the several angles of the hexagonal prisms" (Darwin, 1859, p. 255). The instinct operates without conscious understanding of its adaptive logic - yet it manifests in observable behavior that interacts with environmental feedback, affects survival and reproduction, and remains subject to further modification through selection (see Papineau, 2005).

Darwin illustrates via pointing to canine evolution: "When the first tendency to point was once displayed, methodical selection and the inherited effects of compulsory training in each successive generation would soon complete the work; and unconscious selection is still at work, as each man tries to procure, without intending to improve the breed, dogs which will stand and hunt best" (Darwin, 1859, p. 241). The pointing instinct is modified through unconscious selection - hunters prefer dogs that point well without intending to breed improvement - yet the instinct itself manifests in observable behavior that hunters consciously evaluate and respond to. The unconscious modification produces conscious-level outcomes; the instinct that "does not know itself" nonetheless operates in the arena of conscious perception and evaluation (cf. Bargh & Morsella, 2008).

1.7: Theoretical Synthesis: The Evolutionary Necessity of Unconscious-to-Conscious Integration

Darwin's multifaceted deployment of the "unconscious" consistently describes processes that begin without deliberate intention but produce effects that interact with conscious-level phenomena. Unconscious selection generates observable breed modifications; correlated growth produces phenotypic changes available for conscious evaluation; naturalists' unconscious methodology surfaces in their published classifications; unconscious competition shapes the ecological world organisms consciously navigate; instincts manifest in observable behavior subject to further selection (see Dehaene et al., 2006; Baars, 2002).

This pattern supports the central theoretical claim of the current paper. A singularly subliminal module - processing that never reaches or influences conscious-level phenomena - lacks the feedback mechanisms necessary for adaptive modification (cf. Dennett, 1995). It could not support skill acquisition, problem-solving, social engagement, or behavioral adjustment, because its outputs would never enter the arena where learning, evaluation, and modification occur. Darwin's unconscious, by contrast, continuously feeds into observable outcomes subject to further selection, whether by human breeders, natural environments, or the organisms themselves. The intelligent and progressively adaptive unconscious mind, with conscious interactions that Valsiner (2019) identifies may be not merely a by-product of evolution but a causal principle underlying adaptive modification itself (see also Reber, 1992).

Darwin thus anticipates - over a century and a half in advance - the distinction between subliminality and the unconscious mind that contemporary research must recover. His "unconscious" is not a hidden realm sealed off from awareness but rather the absence of deliberate planning in processes whose effects eventually surface into conscious-level reality. Evolution, on this account, could not have proceeded through singularly subliminal processes; it required the unconscious-to-conscious integration that Darwin consistently emphasizes and exemplifies (cf. Shevrin & Dickman, 1980; Bargh & Morsella, 2008).

References

Baars, B. J. (1997). In the theater of consciousness: The workspace of the mind. Oxford University Press. <https://doi.org/10.1093/acprof:oso/9780195102659.001.1>

Baars B. J. (2002). The conscious access hypothesis: origins and recent evidence. Trends in cognitive sciences, 6(1), 47–52. <https://doi.org/10.1016/s1364-6613(00)01819-2>

Bargh, J. A., & Morsella, E. (2008). The unconscious mind. Perspectives on Psychological Science, 3(1), 73–79. <https://doi.org/10.1111/j.1745-6916.2008.00064.x>

Cosmides, L., & Tooby, J. (1994). Origins of domain specificity: The evolution of functional organization. In L. A. Hirschfeld & S. A. Gelman (Eds.), Mapping the mind: Domain specificity in cognition and culture (pp. 85–116). Cambridge University Press. <https://doi.org/10.1017/CBO9780511752902.005>

Darwin, C. (1859). *On the origin of species by means of natural selection*. John Murray.

Darwin, C. (1868). *The variation of animals and plants under domestication* (Vols. 1-2). John Murray.

Dehaene, S., Changeux, J. P., Naccache, L., Sackur, J., & Sergent, C. (2006). Conscious, preconscious, and subliminal processing: a testable taxonomy. Trends in cognitive sciences, 10(5), 204–211. <https://doi.org/10.1016/j.tics.2006.03.007>

Dennett, D. C. (1995). Darwin's dangerous idea: Evolution and the meanings of life. Simon & Schuster.

Diamond, J. Evolution, consequences and future of plant and animal domestication. Nature 418, 700–707 (2002). <https://doi.org/10.1038/nature01019>

Gregory, T.R. Artificial Selection and Domestication: Modern Lessons from Darwin’s Enduring Analogy. Evo Edu Outreach 2, 5–27 (2009). <https://doi.org/10.1007/s12052-008-0114-z>

Harlan, J. R. (1992). Crops and man (2nd ed.). American Society of Agronomy.

Heiser, C.B. Aspects of unconscious selection and the evolution of domesticated plants. Euphytica 37, 77–81 (1988). <https://doi.org/10.1007/BF00037227>

Hodge, M. J. S. (1977). The structure and strategy of Darwin's "long argument." British Journal for the History of Science, 10(3), 237-246. <https://doi.org/10.1017/S0007087400015685>

Lennox, J. G. (2005). Darwin's methodological evolution. Journal of the History of Biology, 38(1), 85-99.

Mayr, E. (1982). The growth of biological thought: Diversity, evolution, and inheritance. Harvard University Press.

Oatley, K. (2019). The Human Unconscious in Evolution. Psychological Inquiry, 30(2), 76–78. <https://www.jstor.org/stable/48539268>

Papineau, David. (2005). Social learning and the Baldwin effect. 10.4324/9780203012918.

Pigliucci, M. (2001). Phenotypic plasticity: Beyond nature and nurture. Johns Hopkins University Press.

Reber, A. S. (1992). An evolutionary context for the cognitive unconscious. Philosophical Psychology, 5(1), 33–51. <https://doi.org/10.1080/09515089208573042>

Richards, R. J. (1987). Darwin and the emergence of evolutionary theories of mind and behavior. University of Chicago Press.

Secord, J. A. (1981). Nature's fancy: Charles Darwin and the breeding of pigeons. Isis, 72(2), 162-186.

Shevrin, H., & Dickman, S. (1980). The psychological unconscious: A necessary assumption for all psychological theory? American Psychologist, 35(5), 421–434. <https://doi.org/10.1037/0003-066X.35.5.421>

Sloan, P. R. (2019). Darwin: From the Origin of Species to the Descent of Man. In E. N. Zalta (Ed.), The Stanford encyclopedia of philosophy (Winter 2020 ed.). Stanford University.

West-Eberhard, M. J. (2003). Developmental plasticity and evolution. Oxford University Press.

Zohary, D. (2004). Unconscious selection and the evolution of domesticated plants. Economic Botany, 58(1), 5-10.

1. Experimental Methods and Paradigms for Assessing the Unconscious

2.1: Methods and their Implications

Researching unconscious processing presents a deceptively simple question: How can one demonstrate that the mind processes information without the person being aware of it? This question has driven decades of experimental work and theoretical debate and remains one of the most challenging problems in cognitive science today. At the core of this research is a key methodological challenge. Researchers need to show that awareness was absent; however, proving something doesn’t exist is difficult at best. A participant might have had a brief moment of awareness that they did not report. At the same time, researchers must prove that the stimulus still influences Behavior or brain activity. Any observed effect could be attributed to weak or unclear awareness rather than true unconscious processing (Holender, 1986). This challenge affects every study in the field and has driven the creation of more advanced measurement methods. Researchers have developed three main approaches to establish that awareness was absent. The objective threshold approach uses forced-choice tasks in which participants must guess which stimulus appeared. If accuracy equals chance performance (d’ =0; A’/A’’ = .5; A = 0; see Zhang & Mueller, 2005), researchers infer that the stimulus did not reach conscious access. The graded awareness approach, exemplified by the Perceptual Awareness Scale (Ramsøy & Overgaard, 2004), requires participants to rate their experience on a four-point scale ranging from "no experience" to "clear experience." This approach captures partial awareness that binary measures would miss. The Bayesian approach directly quantifies evidence for the null hypothesis. A Bayes Factor greater than three provides positive evidence that awareness was genuinely absent rather than simply undetected by an underpowered measure (Dienes, 2015). Three categories of evidence demonstrate that processing occurred despite absent awareness. Behavioral evidence includes faster responses to primed targets, shifted preferences toward previously exposed stimuli, and advantageous decisions made before participants can articulate why they are choosing as they do (Bechara et al., 1997). Neural evidence tracks processing through time. Early components such as P1 and N1, occurring within 80 to 150 milliseconds, remain intact for stimuli that participants report not seeing. Semantic processing, indexed by the N400 component at approximately 400 milliseconds, can survive attentional manipulations. However, the P300 component, occurring at 300 milliseconds and beyond, appears only for consciously perceived stimuli. Physiological evidence, including skin conductance responses, pupil dilation, and heart rate responses, can reveal emotional or cognitive processing independently of verbal report. Researchers use four main strategies to render stimuli unavailable to conscious report. Visual disruption involves presenting stimuli very briefly, typically 16 to 50 milliseconds, followed immediately by a pattern mask (Dehaene et al., 1998). Alternatively, continuous flash suppression presents dynamic Mondrian patterns to one eye, which suppresses a static image presented to the other eye for periods lasting several minutes (Tsuchiya & Koch, 2005). Attentional disruption exploits the attentional blink phenomenon. When participants identify one target in a rapid serial visual presentation stream, their ability to detect a second target presented 200 to 500 milliseconds later is temporarily impaired (Raymond et al., 1992). Implicit learning paradigms present participants with structured sequences or rule-governed stimuli. Participants demonstrate learning through improved performance but cannot articulate what they have learned (Nissen & Bullemer, 1987). Lesion studies examine patients with damage to the primary visual cortex who demonstrate blindsight, responding appropriately to stimuli in their blind field that they report not seeing (Weiskrantz, 1986). A central debate in this topic revolves around how much processing can occur without awareness. Solid evidence supports unconscious processing of basic visual features, emotional valence, and motor preparation. Mixed evidence exists for more complex processing, including word meaning, face identity, and category membership, with results varying substantially across paradigms and laboratories (Kouider & Dehaene, 2007). Little evidence supports unconscious processing of novel arbitrary associations, complex rules, or executive control functions. This debate remains contentious partly because different paradigms and awareness measures yield systematically different answers about what can be processed without awareness. Three major theoretical frameworks attempt to explain why some processing becomes conscious while other processing does not. Global Workspace Theory proposes that consciousness corresponds to information being broadcast widely across the brain (Dehaene & Changeux, 2011). Unconscious processing remains local and modular, confined to specialized processors. Conscious processing involves ignition of a widespread prefrontal-parietal network that makes information globally available for reports, memory encoding, and flexible Behavioral control. Higher-Order Theory proposes that consciousness requires not merely first-order processing but also meta-awareness and meta-cognition (for a review on these terms, see Lau & Rosenthal, 2011). One must not only process information but also represent the fact that one is processing it. This framework explains blindsight as intact first-order processing without the accompanying higher-order representation that would render it conscious. Predictive Processing frameworks propose that consciousness corresponds to the brain's current best prediction about the causes of sensory input. Unconscious processing involves prediction errors that have not yet been integrated into the global predictive model. Finally, understanding unconscious processing has been suggested to relate to several clinical conditions. In anxiety disorders, it is suggested that the threat detection system shows heightened sensitivity to subliminal danger signals, with the amygdala responding excessively to masked threatening faces even when participants report seeing nothing. In schizophrenia, it is suggested that the boundary between unconscious and conscious processing appears compromised, with automatic associations and internally generated signals intruding inappropriately into awareness (Frith, 1979). In depression, it is suggested that the normal positivity bias in unconscious processing gives way to negativity bias, with implicit self-concept becoming persistently negative. In disorders of consciousness, such as unconsciousness-comma states, it is suggested that neural signatures can reveal covert awareness in patients who show no behavioural response, with profound implications for diagnosis, prognosis, and ethical decision-making (Owen et al., 2006).

References

Bechara, A., Damasio, H., Tranel, D., & Damasio, A. R. (1997). Deciding advantageously before knowing the advantageous strategy. Science (New York, N.Y.), 275(5304), 1293–1295. <https://doi.org/10.1126/science.275.5304.1293>

Dehaene, S., & Changeux, J. P. (2011). Experimental and theoretical approaches to conscious processing. Neuron, 70(2), 200–227. <https://doi.org/10.1016/j.neuron.2011.03.018>

Dehaene, S., Naccache, L., Le Clec'H, G., Koechlin, E., Mueller, M., Dehaene-Lambertz, G., van de Moortele, P. F., & Le Bihan, D. (1998). Imaging unconscious semantic priming. Nature, 395(6702), 597–600. <https://doi.org/10.1038/26967>

Dienes, Zoltan. (2015). How Bayesian statistics are needed to determine whether mental states are unconscious. 10.1093/acprof:oso/9780199688890.003.0012.

Frith C. D. (1979). Consciousness, information processing and schizophrenia. The British journal of psychiatry : the journal of mental science, 134, 225–235. <https://doi.org/10.1192/bjp.134.3.225>

Holender, D. (1986). Semantic activation without conscious identification in dichotic listening, parafoveal vision, and visual masking: A survey and appraisal. Behavioral and Brain Sciences, 9(1), 1–23. doi:10.1017/S0140525X00021269

Kouider, S., & Dehaene, S. (2007). Levels of processing during non-conscious perception: a critical review of visual masking. Philosophical transactions of the Royal Society of London. Series B, Biological sciences, 362(1481), 857–875. <https://doi.org/10.1098/rstb.2007.2093>

Lau, H., & Rosenthal, D. (2011). Empirical support for higher-order theories of conscious awareness. Trends in cognitive sciences, 15(8), 365–373. <https://doi.org/10.1016/j.tics.2011.05.009>

Nissen, M. J., & Bullemer, P. (1987). Attentional requirements of learning: Evidence from performance measures. Cognitive Psychology, 19(1), 1–32. <https://doi.org/10.1016/0010-0285(87)90002-8>

Owen, A. M., Coleman, M. R., Boly, M., Davis, M. H., Laureys, S., & Pickard, J. D. (2006). Detecting awareness in the vegetative state. Science (New York, N.Y.), 313(5792), 1402. <https://doi.org/10.1126/science.1130197>

Ramsøy, T. Z., & Overgaard, M. (2004). Introspection and subliminal perception. Phenomenology and the Cognitive Sciences, 3(1), 1–23. <https://doi.org/10.1023/B:PHEN.0000041900.30172.e8>

Raymond, J. E., Shapiro, K. L., & Arnell, K. M. (1992). Temporary suppression of visual processing in an RSVP task: an attentional blink? . Journal of experimental psychology. Human perception and performance, 18(3), 849–860. <https://doi.org/10.1037//0096-1523.18.3.849>

Tsuchiya, N., & Koch, C. (2005). Continuous flash suppression reduces negative afterimages. Nature neuroscience, 8(8), 1096–1101. <https://doi.org/10.1038/nn1500>

Shevrin, H. (1997). Commentaries. Journal of the American Psychoanalytic Association, 45(3), 746-752. <https://doi.org/10.1177/00030651970450031209>

Zhang, J., & Mueller, S., T. (2005). A note on ROC analysis and non-parametric estimate of sensitivity. *Psychometrika*, *70*, 203–212.

2.2.: Experimental Paradigms, Methods and their Implications for the Unconscious Mind

The confirmation of evidence for unconscious processing varies considerably across paradigms, and several influential findings have faced challenges. Motor priming effects from masked stimuli have been replicated consistently across laboratories. Researchers using masked primes have demonstrated reliable response preparation effects, with lateralized readiness potentials indicating that the motor system begins preparing responses to stimuli that participants cannot report (Dehaene et al., 1998). Basic feature processing, including orientation, color, and motion, survives masking and attentional manipulation with reasonable positive consistency. Documentation for emotional valence extraction from masked faces has replicated across multiple paradigms, with amygdala activation to unseen threatening faces observed in both neuroimaging and lesion studies. The Iowa Gambling Task, once considered strong evidence for unconscious decision-making, has faced substantial criticism. Maia and McClelland (2004) demonstrated that participants who showed anticipatory skin conductance responses could, when properly questioned, have considerable knowledge about deck contingencies. This finding suggests that the original interpretation conflated poor metacognitive access with genuinely unconscious knowledge. Semantic priming from masked words remains controversial. While some studies demonstrate category-level priming, effect sizes are typically small and highly sensitive to stimulus parameters. Kouider and Dehaene (2007) concluded that semantic processing of masked words, when it occurs, is partial and degraded rather than equivalent to conscious semantic access. Several early demonstrations of unconscious perception have not survived methodological scrutiny. Studies claiming subliminal persuasion effects have consistently failed to replicate under controlled conditions. The "mere exposure" effect, while robust for supraliminal stimuli, shows inconsistent results when stimuli are rendered genuinely invisible rather than merely degraded. Claims of unconscious arithmetic and unconscious reading of sentences have been challenged by studies showing that minimal awareness, captured by sensitive measures like the Perceptual Awareness Scale, can account for apparent unconscious effects (Sand & Nilsson, 2016). A recurring issue across paradigms is whether null awareness truly reflects absent awareness or merely insensitive measurement. When researchers using the Perceptual Awareness Scale re-examine stimuli previously classified as "unseen" using binary measures, they often find that participants report "brief glimpses" rather than complete absence of experience. This raises the possibility that some proportion of reported unconscious processing effects reflect processing of degraded but not absent conscious representations. The field has moved toward a more conservative position than was common in the 1990s and early 2000s. Researchers now generally accept that basic perceptual and affective processing can occur without reportable awareness, but claims for unconscious semantic processing, unconscious decision-making, and unconscious executive function face continued skepticism. The most defensible position holds that unconscious processing is real but limited in scope, primarily supporting early perceptual analysis and simple associative responses rather than the complex cognition that some early theorists proposed. Three major theoretical frameworks attempt to explain why some processing becomes conscious while other processing does not. Global Workspace Theory proposes that consciousness corresponds to information being broadcast widely across the brain (Dehaene & Changeux, 2011). Unconscious processing remains local and modular, confined to specialized processors. Conscious processing involves ignition of a widespread prefrontal-parietal network that makes information globally available for report, memory encoding, and flexible behavioral control. Higher-Order Theory proposes that consciousness requires not merely first-order processing but meta-awareness (Lau & Rosenthal, 2011). One must not only process information but also represent the fact that one is processing it. This framework explains blindsight as intact first-order processing without the accompanying higher-order representation that would render it conscious. The Predictive Processing Frameworks propose that consciousness corresponds to the brain's current best prediction about the causes of sensory input. Unconscious processing involves prediction errors that have not yet been integrated into the global predictive model. Overall, the diversity of methods, and the frequent error of equating subliminality with workings of the unconscious mind in topical experimental paradigms (Bargh & Morsella, 2008), shows that although the concept oft the unconscious mind seems to confer higher conceptual and ecological validity, compared to the conflicted notion of subliminality, we have not yet accomplished the implementation of dedicated paradigms for its exploration.

References

Bargh, J. A., & Morsella, E. (2008). The unconscious mind. Perspectives on Psychological Science, 3(1), 73–79. <https://doi.org/10.1111/j.1745-6916.2008.00064.x>

Bechara, A., Damasio, H., Tranel, D., & Damasio, A. R. (1997). Deciding advantageously before knowing the advantageous strategy. Science (New York, N.Y.), 275(5304), 1293–1295. <https://doi.org/10.1126/science.275.5304.1293>

Dehaene, S., & Changeux, J. P. (2011). Experimental and theoretical approaches to conscious processing. Neuron, 70(2), 200–227. <https://doi.org/10.1016/j.neuron.2011.03.018>

Dehaene, S., Naccache, L., Le Clec'H, G., Koechlin, E., Mueller, M., Dehaene-Lambertz, G., van de Moortele, P. F., & Le Bihan, D. (1998). Imaging unconscious semantic priming. Nature, 395(6702), 597–600. <https://doi.org/10.1038/26967>

Dienes, Zoltan. (2015). How Bayesian statistics are needed to determine whether mental states are unconscious. <https://doi.org/10.1093/acprof:oso/9780199688890.003.0012>

Frith C. D. (1979). Consciousness, information processing and schizophrenia. The British journal of psychiatry : the journal of mental science, 134, 225–235. <https://doi.org/10.1192/bjp.134.3.225>

Holender, D. (1986). Semantic activation without conscious identification in dichotic listening, parafoveal vision, and visual masking: A survey and appraisal. Behavioral and Brain Sciences, 9(1), 1–23. <https://doi.org/10.1017/S0140525X00021269>

Kouider, S., & Dehaene, S. (2007). Levels of processing during non-conscious perception: a critical review of visual masking. Philosophical transactions of the Royal Society of London. Series B, Biological sciences, 362(1481), 857–875. <https://doi.org/10.1098/rstb.2007.2093>

Lau, H., & Rosenthal, D. (2011). Empirical support for higher-order theories of conscious awareness. Trends in cognitive sciences, 15(8), 365–373. <https://doi.org/10.1016/j.tics.2011.05.009>

Maia, T. V., & McClelland, J. L. (2004). A reexamination of the evidence for the somatic marker hypothesis: what participants really know in the Iowa gambling task. Proceedings of the National Academy of Sciences of the United States of America, 101(45), 16075–16080. <https://doi.org/10.1073/pnas.0406666101>

Nissen, M. J., & Bullemer, P. (1987). Attentional requirements of learning: Evidence from performance measures. Cognitive Psychology, 19(1), 1–32. <https://doi.org/10.1016/0010-0285(87)90002-8>

Owen, A. M., Coleman, M. R., Boly, M., Davis, M. H., Laureys, S., & Pickard, J. D. (2006). Detecting awareness in the vegetative state. Science (New York, N.Y.), 313(5792), 1402. <https://doi.org/10.1126/science.1130197>

Ramsøy, T. Z., & Overgaard, M. (2004). Introspection and subliminal perception. Phenomenology and the Cognitive Sciences, 3(1), 1–23. <https://doi.org/10.1023/B:PHEN.0000041900.30172.e8>

Raymond, J. E., Shapiro, K. L., & Arnell, K. M. (1992). Temporary suppression of visual processing in an RSVP task: an attentional blink? . Journal of experimental psychology. Human perception and performance, 18(3), 849–860. <https://doi.org/10.1037//0096-1523.18.3.849>

Sand, A., & Nilsson, M. E. (2016). Subliminal or not? Comparing null-hypothesis and Bayesian methods for testing subliminal priming. Consciousness and Cognition: An International Journal, 44, 29–40. <https://doi.org/10.1016/j.concog.2016.06.012>

Tsuchiya, N., & Koch, C. (2005). Continuous flash suppression reduces negative afterimages. Nature neuroscience, 8(8), 1096–1101. <https://doi.org/10.1038/nn1500>

Shevrin, H. (1997). Commentaries. Journal of the American Psychoanalytic Association, 45(3), 746-752. <https://doi.org/10.1177/00030651970450031209>

1. “A Quantitative Disentanglement”: Responses, No Responses, and Null Responses

“NHST is a statistical method that, employing, in this area, an analysis called a one-sample t-test, could allow a researcher to conduct a comparison between the participants’ detection responses and mere-guess chance-level detection responses (e.g., 50%), such as responses to imperceptible stimuli or, in more colloquial terms, responses that we would expect from a blind individual. This method could provide evidence for whether a researcher could obtain significant or non-significant results, meaning whether they were able or whether they were not able to reject the null hypothesis that the participants’ detection performance was not statistically different to chance. In case of non-significant results, such as failing to reject the null, the out­comes of this analysis were erroneously interpreted to show significance for proxim­ity, such as evidence for the null (Dienes, 2015, 2016). In this statistical analysis, we misinterpreted failing to reject the null hypothesis, that there are no significant differences between the participants’ performance and chance, with providing direct evidence that confirm the null hypoth­esis, that the participants’ performance and chance were statistically proximate (see Dienes, 2016). Moreover, we already discussed that NHST cannot provide us with evidence that perception is at-chance. This method can only provide us with evidence that we can reject the null hypothesis that perception was not different to chance. Bayesian analysis can provide direct evidence for chance-level perception. Bayesian analysis requires a lower (LB) and an upper bound (UB). These bounds are called credible intervals. They stand for the lowest and highest values within which we can define a meaningful range for exploring whether the participants’ perception was proxi­mate to chance-level perception (e.g., A chance-level = .5; Lower Bound (LB) = .4 or .45; Upper Bound (UP) = .6 or .55; see Tsikandilakis et al., 2020). The credible intervals can be based on previous research findings, or a theoretically-driven ratio­nale (Schönbrodt & Wagenmakers, 2018) or based on pre-determined study-specific expectancies, such as end-user requirement characteristics, or study-specific mini­ mum effect sizes of interest (e.g., η^2^_p_ ≥ .01 or Cohen’s d ≥ .2; for a dedicated review, see Dienes et al., 2018). Bayesian inference requires the standard error of the population sample, and a simple deduction of the sample mean from chance to provide a Bayes Factor (BF; see Dienes, 2014). The BF shows at a BF < .33 direct evidence for the likelihood of the data being observed if the null hypothesis is true (i.e., chance-level detection perfor­mance), at .33 < BF < 3 that the results are inconclusive, and at BF > 3 evidence for the likelihood of the data being observed under the alternate hypothesis, that partici­pants’ detection performance was substantially different to chance (Dienes, 2015).”

This passage is adapted with journal permissions from Tsikandilakis and colleagues (2025; pp. 7-12).

References

Dienes, Z. (2014). Using Bayes to get the most out of non-significant results. *Frontiers in psychology*, *5*, 781-798.

Dienes, Z. (2015). How Bayesian statistics are needed to determine whether mental states are unconscious. *Behavioral methods in consciousness research*, 199-220.

Dienes, Z. (2016). How Bayes factors change scientific practice. *Journal of Mathematical Psychology*, *72*, 78-89.

Schönbrodt, F. D., & Wagenmakers, E. J. (2018). Bayes factor design analysis: Planning for compelling evidence. *Psychonomic bulletin & review*, *25*(1), 128-142.

Tsikandilakis, M., Bali, P., Derrfuss, J., & Chapman, P. (2020). “I can see you; I can feel it; and vice-versa”: consciousness and its relation to emotional physiology. *Cognition and Emotion*, *34*(3), 498–510.

Tsikandilakis, M., Bali, P., Pasachidou, V. M., Toranzos, R. L., Szczesniak, K., Mével, P. A., ... & Milbank, A. (2025). A Re-Exploration of our Unconscious: What We Have Come To Unmask; What Still Lies Beneath. *Integrative Psychological and Behavioral Science*, *59*(4), 1-36.
